# Synthesis and Characterization of Nitrogen-doped Carbon Nanotubes Derived from g-C_3_N_4_

**DOI:** 10.3390/ma13061349

**Published:** 2020-03-17

**Authors:** Klaudia Maślana, Ryszard J. Kaleńczuk, Beata Zielińska, Ewa Mijowska

**Affiliations:** Nanomaterials Physicochemistry Department, Faculty of Chemical Technology and Engineering, West Pomeranian University of Technology, Piastow Av. 45, 70-311 Szczecin, Poland; mk30511@zut.edu.pl (K.M.); rk@zut.edu.pl (R.J.K.); emijowska@zut.edu.pl (E.M.)

**Keywords:** graphitic carbon nitride, nickel oxides, nitrogen-doped carbon nanotubes, CVD process

## Abstract

Here, nitrogen-doped carbon nanotubes (CNT-N) were synthesized using exfoliated graphitic carbon nitride functionalized with nickel oxides (ex-g-C_3_N_4_-NixO_y_). CNT-N were produced at 900 °C in two steps: (1) ex-g-C_3_N_4_-Ni_x_O_y_ reduction with hydrogen and (2) ethylene assisted chemical vapor deposition (CVD). The detailed characterization of the produced materials was performed via atomic force microscopy (AFM), transmission electron microscopy (TEM), Raman spectroscopy, X-ray diffraction (XRD) and thermogravimetric analysis (TGA). The possible mechanism of nanotubes formation is also proposed.

## 1. Introduction

The discovery of diverse nanocarbon allotropes and its nanocomposites has inspired scientists for a range of potential applications. Here, for many years, carbon nanotubes have received huge scientific interest due to their unusual features, such as unique atomic structure, high surface, remarkable mechanical, electrical and thermal properties. The modification of carbon nanotubes (CNTs) by attaching functional groups to the surface and doping with heteroatoms or metal/metal oxide nanoparticles is the most promising strategy with respect to their potential application. For example, the doping of CNTs with nitrogen makes defects that alter the chemical properties of CNTs and creates a path to applications.

It is well known that the properties of solid materials are attributed to surface morphology. Thus, materials have to be modified prior to applications to improve their specific properties [1,2,3,4,5,6]. The surface modification of materials can be done by two main paths: *(i)* by attaching functional groups [7,8,9] and *(ii)* by introducing heteroatoms (e.g., nitrogen, boron, phosphorus, sulfur) in the molecular structure of materials [10,11,12]. From all of the heteroatoms, nitrogen is the one of the most investigated because it is sustainable for carbon replacing due to the fact that N and C atoms possess similar atomic radius and are chemically relatively easy to replace [13,14]. Furthermore, nitrogen possesses one electron more than carbon, which is desirable for electrochemical reactions such as the oxygen evolution reaction (OER) [15]. Nitrogen doping may also affect electron conductivity, electron-donor properties and chemical stability of the host material due to the modification of the electronic and crystal structure [16]. Moreover, the incorporation of nitrogen into the structure of molecules modifies their geometric properties, which results in an increase in the number of active sites and improves the interaction between the carbon structure and absorbate [17].

Nitrogen-doped carbon nanotubes have found application in many various fields, such as electrochemistry, energy storage, CO_2_ adsorption, Li-ion batteries and others [18,19,20,21,22]. In this context, Wu et al. [23] demonstrated that N-doped CNT enhanced the energy storage capacity and improved properties of produced batteries. Ariharan et al. [17] revealed that nitrogen-doped carbon nanotubes showed a higher hydrogen storage capacity of ~ 2 wt% total adsorption, which is higher compared to other nitrogen-enriched carbon nanostructures. Another team, Liu et al. [24], reported that nitrogen-doped carbon nanotubes and graphitic carbon nitride nanocomposites exhibit an excellent ability to photocatalytic hydrogen evolution rate of 1208 μmol g^−1^h^−1^. In addition to applications related to electrochemistry, nitrogen-doped carbon nanotubes have been used for production sensors [25] or organic pollutant removal [26].

Various preparation methods have been employed to obtain nitrogen-doped carbon nanotubes. The most widely used method of preparing nitrogen-doped carbon nanotubes is chemical vapor deposition [27,28]. One of the most significant advantages of this method is its simplicity, which is the reason for its widespread use. However, N-doped CNT are obtained by other methods as well, such as in situ synthesis and post nitridation treatment [29], catalytic pyrolysis of dimethylformamide [30] and plasma treatment [31].

It is well known that ethylene plays the most important role during the chemical vapor deposition process. It is the main source of carbon, which in this process is necessary for the growth of carbon nanotubes. During the production of single-walled carbon nanotubes and multi-walled carbon nanotubes, hydrocarbons such as ethylene, acetylene, benzene or methane have been decomposed into single carbon atoms or linear dimers/trimers. This step takes place on the surface of metallic catalysts and results in CNT growth [32,33]. The process usually takes place in the temperature range of 500–1000 °C [34].

In this work, we present a novel route to prepare nitrogen-doped carbon nanotubes (CNT-N) from graphitic carbon nitride functionalized with nickel oxides (ex-g-C_3_N_4_-Ni_x_O_y_) by the chemical vapor deposition (CVD) method. We also propose the mechanism of reshaping of the flake-like structure into the tubular form.

## 2. Details Experimental

### 2.1. Materials and Procedures

Melamine (C_3_H_6_N_6_, 99.0%) and nickel (II) acetate tetrahydrate (Ni(OCOCH_3_)_2_·4H_2_O, ≥ 99.0%) were purchased from Sigma Aldrich (St. Louis, MI, USA).

#### 2.1.1. Synthesis of Exfoliated Graphitic Carbon Nitride

Bulk graphitic carbon nitride (g-C_3_N_4_) was synthesized via thermal polycondensation of melamine. The process was carried out in muffle furnace at 550 °C for 2 h with a heating rate of 2 °C/min in air atmosphere. Next, the obtained yellow product was exfoliated by sonication using Tip-ultra-sonicator (500 W, Sonics VC505, Sonics & Materials Inc., Newtown, USA). Here, a certain amount of the bulk g-C_3_N_4_ was mixed with isopropyl alcohol and sonicated for 24 h. The final powdered product was obtained by evaporation of the solvent at 50 °C. The exfoliated sample was denoted as ex-g-C_3_N_4_.

#### 2.1.2. Synthesis of Functionalized Exfoliated Graphitic Carbon Nitride with Nickel Oxides

In a typical synthesis of the procedure, ex-g-C_3_N_4_ and Ni(OCOCH_3_)_2_·4H_2_O in a weight ratio of 1:1 were mixed in isopropyl alcohol by sonication for 3 h. Then, the obtained dispersion was stirred until the solvent evaporated. Finally, the product was annealed at 440 °C for 10 min. with a heating rate of 6 °C/min in a vacuum.

#### 2.1.3. Synthesis of Nitrogen-doped Carbon Nanotubes

Exfoliated graphitic carbon nitride functionalized with nickel oxides (ex-g-C_3_N_4_-Ni_x_O_y_) was placed in the ceramic boat and introduced into the tubular furnace. The furnace was heated under flowing nitrogen up to 900 °C. When the temperature was reached, hydrogen was introduced (100 sccm) for 3 h. Afterward, the hydrogen supply was cut off and ethylene was introduced to the furnace for 10 min. Subsequently, the furnace was cooled down to room temperature. The as-obtained product was denoted as CNT-N.

### 2.2. Characterization

The morphology of the obtained samples was investigated by transmission electron microscopy with energy dispersive X-ray (EDX) spectroscopy (Tecnai F20-based at 200 kV accelerating voltage, Thermo Fisher Scientific, Waltham, MA, USA) and scanning electron microscopy (TESCAN, VEGA 3, acquired in the 30 kV acceleration voltage, TESCAN, Brno, Czech Republic). Selected area electron diffraction (SAED) patterns were obtained using TEM microscope (Thermo Fisher Scientific, Waltham, MA, USA). The AFM study was performed using an atomic force microscope (Nanoscope V Multimode 8, Bruker AXS, Mannheim, Germany). X-ray diffraction (XRD) patterns were carried out using an Empyrean (Malvern Panalytical, Malvern, UK) diffractometer using CuKα radiation. Thermogravimetric analysis was carried out using TA-Q600 SDT TA Instrument (TA Instrument, New Castle, DE, USA) under the air and argon atmosphere at ramping rate of 10 °C/min from room temperature to 900 °C. Raman spectra were determined on inVia Raman Microscope (RENISHAW, New Mills Wotton-under-Edge, UK) with an excitation wavelength of 785 nm.

## 3. Results and Discussion

### 3.1. Microscopic Analysis

**Figure 1:**
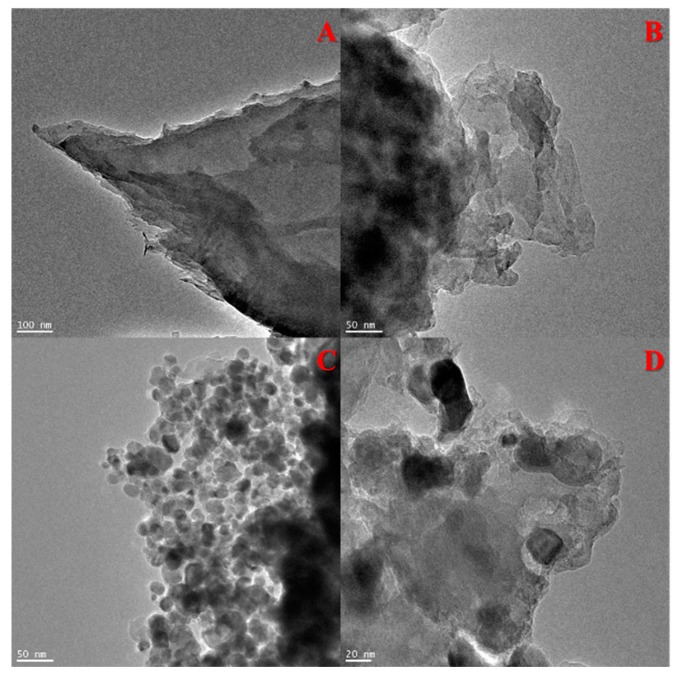
Transmission electron microscopy (TEM) images of (**A**) g-C_3_N_4_, (**B**) ex-g-C_3_N_4_ and (**C**,**D**) ex-g-C_3_N_4_-Ni_x_O_y_.

The morphologies of g-C_3_N_4_, ex-g-C_3_N_4_ and ex-g-C_3_N_4_-Ni_x_O_y_ were studied by transmission electron microscopy. The TEM images of the samples are presented in Figure 1. TEM images of g-C_3_N_4_ and ex-g-C_3_N_4_ (Figure 1A,B) show a visible difference in the thickness of these two samples. Figure 1C and D exhibit that Ni_x_O_y_ nanoparticles were successfully deposited on the flakes of ex-g-C_3_N_4_ (ex-g-C_3_N_4_-Ni_x_O_y_). The average diameter of Ni_x_O_y_ particles is ~32 nm.

**Figure 2:**
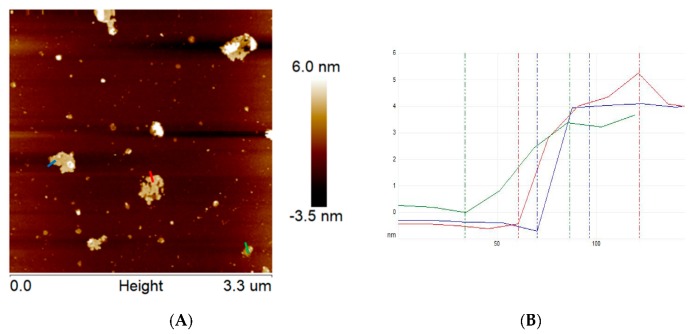
AFM images of ex-g-C_3_N_4_ (**A**) and corresponding high profile (**B**).

Additionally, the topography of the exfoliated g-C_3_N_4_ was also examined via atomic force microscopy (Figure 2). The obtained data indicate that the thickness of ex-g-C_3_N_4_ is ~ 4–5 nm, which means that this sample is composed of ~10–13 layers. According to the fact that bulk graphitic carbon nitride is composed of ~100–170 layers, we can conclude that the exfoliation step was efficient. Based on AFM images, the number of ex-g-C_3_N_4_ layers is reduced by more than 30 times in comparison to the pristine g-C_3_N_4_, which is supported by TEM.

**Figure 3:**
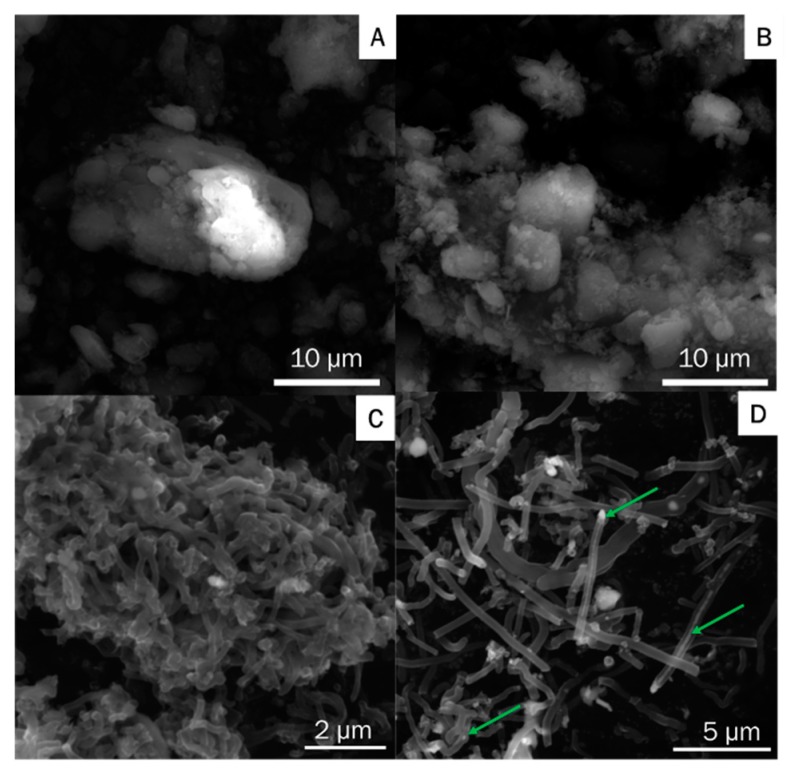
Scanning electron microscopy (SEM) images of (**A**) g-C_3_N_4_, (**B**) ex-g-C_3_N_4_ and (**C**,**D**) CNT-N.

**Figure 4:**
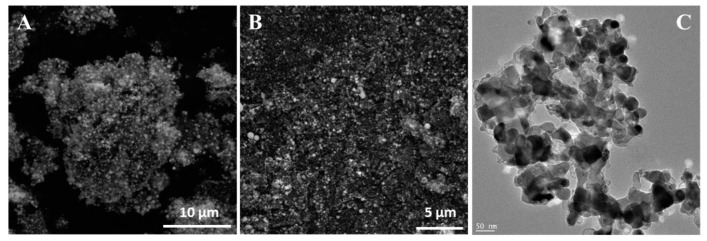
SEM (**A**,**B**) and TEM (**C**) images of g-C_3_N_4_-Ni_x_O_y_ treated with hydrogen at 900 °C.

Figure 3 shows SEM images of g-C_3_N_4_, ex-g-C_3_N_4_ and the sample produced in the process of hydrogen reduction of ex-g-C_3_N_4_-Ni_x_O_y_ and next CVD in ethylene in the presence of reduced ex-g-C_3_N_4_-Ni_x_O_y_ (CNT-N). g-C_3_N_4_ and ex-g-C_3_N_4_ (Figure 3A,B) exhibited aggregated morphology with irregular multiple-layered structures. The morphology of CNT-N (Figure 3C,D) is quite different from that of g-C_3_N_4_ and ex-g-C_3_N_4_. This sample is composed of nanotubular structures. Moreover, nickel residues which served as catalysts in nanotube growth were observed inside the tubes (green arrows in Figure 3D). The obtained nanotubes tend to agglomerate into larger clusters. The reference experiment without ethylene was also performed (Figure 4). This confirmed that ethylene is crucial in the process of nitrogen-doped carbon nanotube formation.

**Figure 5:**
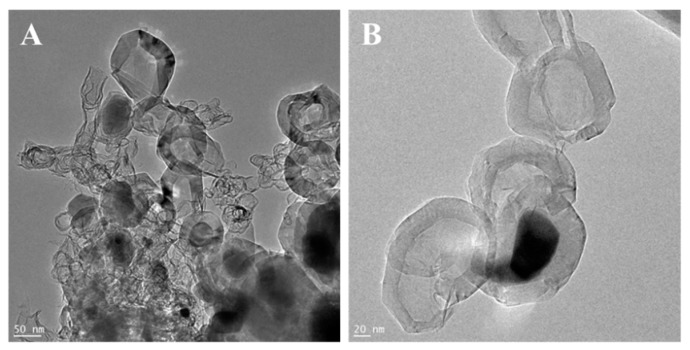
TEM (**A**,**B**) images of g-C_3_N_4_-Ni_x_O_y_ treated with ethylene at 900 °C.

To determine the role of ethylene during tube formation, an additional experiment was performed. In this step, g-C_3_N_4_-Ni_x_O_y_ was heated to 900 °C and then treated with hydrogen for 3 h. In this case, the sample had not been exposed to ethylene. SEM and TEM images of the obtained product are presented in Figure 4. Here, tubular forms did not evolve. Additionally, metal particles agglomerated into larger clusters. A further experiment with g-C_3_N_4_-Ni_x_O_y_ treated only with ethylene (no hydrogen step) at 900 °C was conducted (Figure 5). It revealed that the obtained sample contained mostly onion-like particles. Some of these structures are formed on the surface of metal particles. Detailed TEM investigations revealed a few irregular, deformed tubular structures.

**Figure 6:**
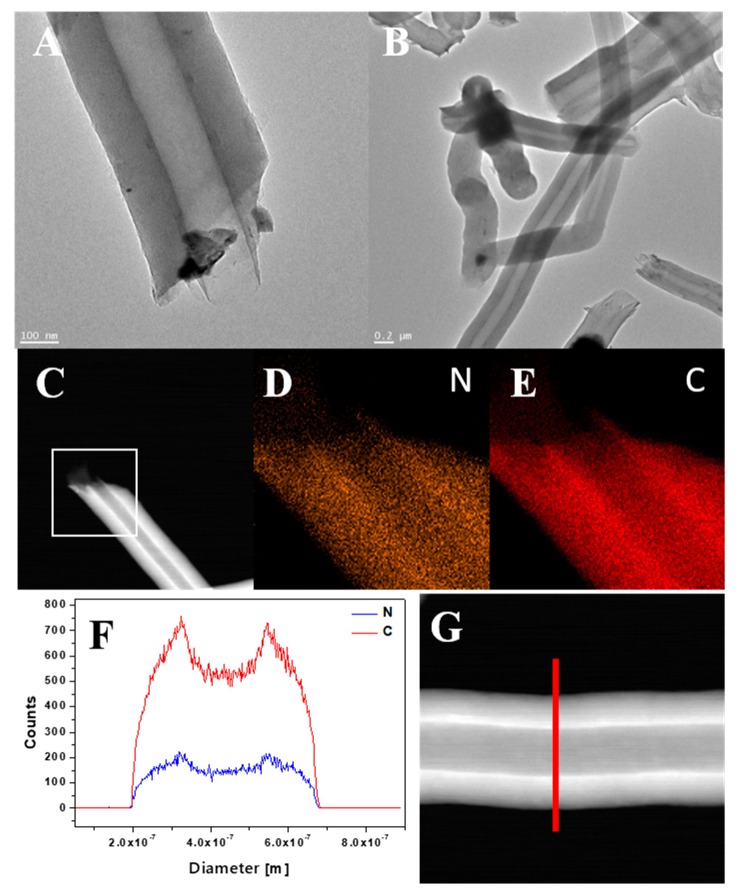
TEM images of CNT-N (**A**,**B**), STEM image of CNT-N (**C**) with corresponding mapping image of nitrogen (**D**) and carbon (**E**), and line profile (**F**) of CNT_N with corresponding STEM image (**G**).

In order to study the morphology and elemental composition of CNT-N in greater detail, TEM, STEM and EDS analyses were performed (Figure 6). TEM images (Figure 6A,B) confirmed that the nanotubes have a diameter in the range of 200–300 nm. The elemental mapping (Figure 6D,E), carried out for the area marked as a white square on the STEM image (Figure 6C), revealed that carbon and nitrogen are the only elements present in the sample. Moreover, both components are homogeneously distributed in nanotubes. Furthermore, the line scan along the direction denoted by the red line in the STEM image (Figure 6G) was carried out and the obtained profiles are presented in Figure 6F. This analysis additionally confirms the chemical composition of CNT-N nanotubes.

**Figure 7:**
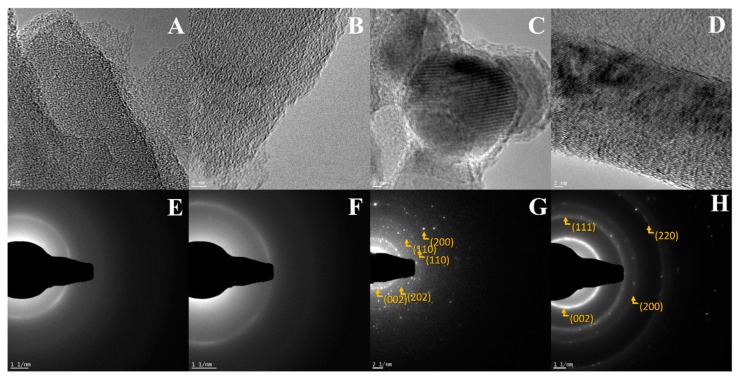
HR-TEM (**A**–**D**) and SAED (**E**–**H**) images of g-C_3_N_4_ (**A**,**E**), ex-g-C_3_N_4_ (**B**,**F**), ex-g-C_3_N_4_-Ni_x_O_y_ (**C**,**G**) and CNT-N (**D**,**H**).

Figure 7 presents HR-TEM and SAED images for g-C_3_N_4_, ex-g-C_3_N_4_, ex-g-C_3_N_4_-Ni_x_O_y_ and CNT-N. For g-C_3_N_4_ and ex-g-C_3_N_4_ crystal fingers are not observed, which indicates that those products are amorphous, which is confirmed by SEAD analysis [35]. ex-g-C_3_N_4__Ni_x_O_y_ and CNT_N exhibit SAED patterns typical for a polycrystalline structure. ex-g-C_3_N_4_-Ni_x_O_y_ shows a few diffraction rings with discrete reflections corresponding to the different orientations of the single phase. The following rings have been characterized: (002) corresponding to nickel particles, (202) to Ni_2_O_3_, (110) to NiO_2_ and (110) to Ni. The pattern of copper (from TEM grid) can also be observed and is marked with (220). This analysis is in full agreement with the XRD analysis, which proved the presence of different nickel compounds in the sample. CNT-N exhibits three clear rings. The first two rings (111) and (200) are typical for nickel nanoparticles. Similar to the previous sample, the rings from copper can be detected.

### 3.2. Spectroscopic, Crystal and Thermogravimetric Studies

**Figure 8:**
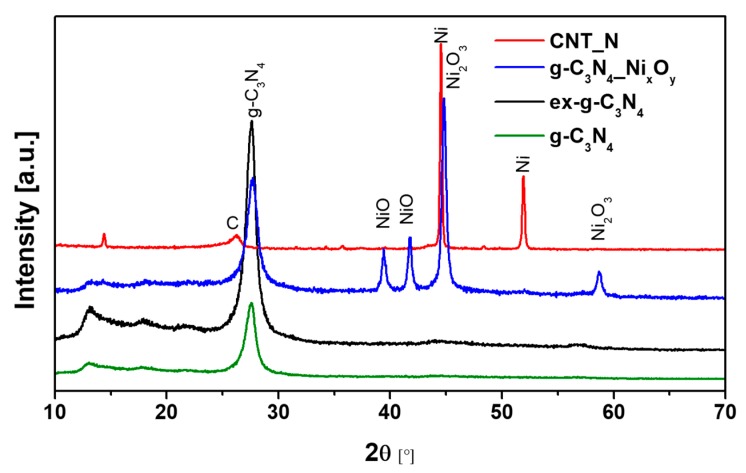
XRD patterns of g-C_3_N_4_, ex-g-C_3_N_4_, ex-g-C_3_N_4_-Ni_x_O_y_ and CNT-N.

XRD patterns of g-C_3_N_4_, ex-g-C_3_N_4_, ex-g-C_3_N_4_-Ni_x_O_y_ and CNT-N samples are shown in Figure 8. The peaks located at 13.1° and 27.56° can be observed for three samples (g-C_3_N_4_, ex-g-C_3_N_4_, ex-g-C_3_N_4_-Ni_x_O_y_) and they are attributed to the presence of graphitic carbon nitride. Peaks at 13.1° and 27.56° correspond to the (100) and (002) crystalline phase, respectively (JCPDS 87–1526). Nickel-functionalized graphitic carbon nitride (ex-g-C_3_N_4_-Ni_x_O_y_) exhibits the presence of different forms of a nickel compound. The diffraction peak at 2θ 44.8° and 58.7° is related to the nickel (III) oxide (JCPDS 14–0481), while two peaks at ~39.4° and ~41.8° belong to nickel (II) oxide (JCPDS 85–1977) [36,37]. During the last stage of CNT-N synthesis, nickel oxide is reduced to the metallic form of nickel and catalyzes the growth of nanotubes. This is confirmed by the disappearance of the peaks from NiO and Ni_2_O_3_ and the appearance of signals characteristic for the metallic form of nickel located at ~44.5° and ~51.9° (JCPDS 87–0712) [38]. Moreover, the diffraction peak at 26.2° observed for CNT-N is assigned to the (002) plane of graphitic carbon.

**Figure 9:**
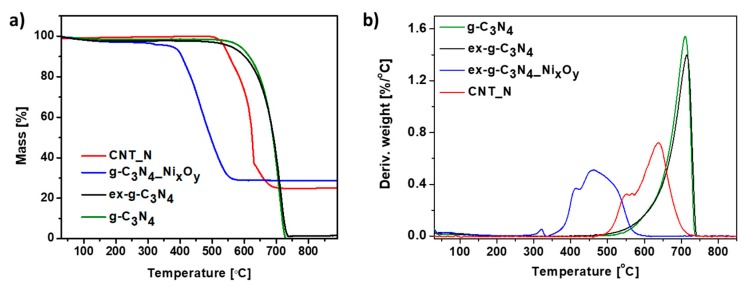
Thermogravimetric (**a**) and derivative thermogravimetric curves (**b**) of g-C_3_N_4_, ex-g-C_3_N_4_, ex-g-C_3_N_4_-Ni_x_O_y_ and CNT-N conducted in air.

Thermogravimetric analysis was carried out in order to determine the thermal properties of the samples. Figure 9 represents the TG (a) and DTG (b) curves of g-C_3_N_4_, ex-g-C_3_N_4_, ex-g-C_3_N_4_-Ni_x_O_y_ and CNT-N heat-treated under air flow. Here, the exfoliation process does not affect the thermal stability of graphitic carbon nitride. For both g-C_3_N_4_ and ex-g-C_3_N_4_, the degradation process starts at about 500 °C and continues until complete decomposition. Functionalization with nickel oxides (ex-g-C_3_N_4_-NixOy) significantly decreased the decomposition temperature to 350 °C. The residue mass after the analysis is ~28.7 wt% and this value is assigned to the amount of nickel compounds in the sample. Two steps can be distinguished on the TGA curve obtained for the CNT-N sample. The first one begins at a temperature of 468 °C and is attributed to the removal of more defected carbon structures. The second one, from a temperature of 565 °C, is related to the degradation of carbon nanotubes. The residual mass is ~24.8 wt% which is the nickel compounds content in the sample.

**Figure 10:**
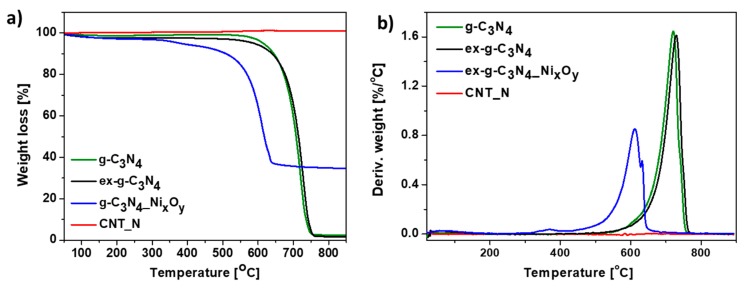
Thermogravimetric (**a**) and derivative thermogravimetric (**b**) curves of g-C_3_N_4_, ex-g-C_3_N_4_, ex-g-C_3_N_4_-Ni_x_O_y_ and CNT-N conducted in Ar.

Additionally, TGA analysis performed in argon atmosphere was conducted. Thermogravimetric (TG) (Figure 9a) and derivative thermogravimetric (DTG) (Figure 9b) curves of g-C_3_N_4_, ex-g-C_3_N_4_, ex-g-C_3_N_4_-Ni_x_O_y_ and CNT-N heat-treated under argon flow are presented in Figure 10. g-C_3_N_4_ and ex-g-C_3_N_4_ are stable up to ~500 °C, either in air or argon atmosphere (see Figure 9 and Figure 10). This means that both g-C_3_N_4_ and ex-g-C_3_N_4_ can endure high temperatures, even in an oxidizing atmosphere. The weight loss at above 500 °C is not due to the oxidation by O_2_, but to the direct thermal decomposition of g-C_3_N_4_ itself [39]. Moreover, the residual mass of ~3 wt% is observed in an inert atmosphere. ex-g-C_3_N_4_-NixOy exhibited different thermal stability in air and argon atmosphere, which was higher in Ar flow. Furthermore, the residual mass after analysis is ~34 wt% (~24.8 wt% in air). The obtained CNT-N is stable in argon atmosphere.

**Figure 11:**
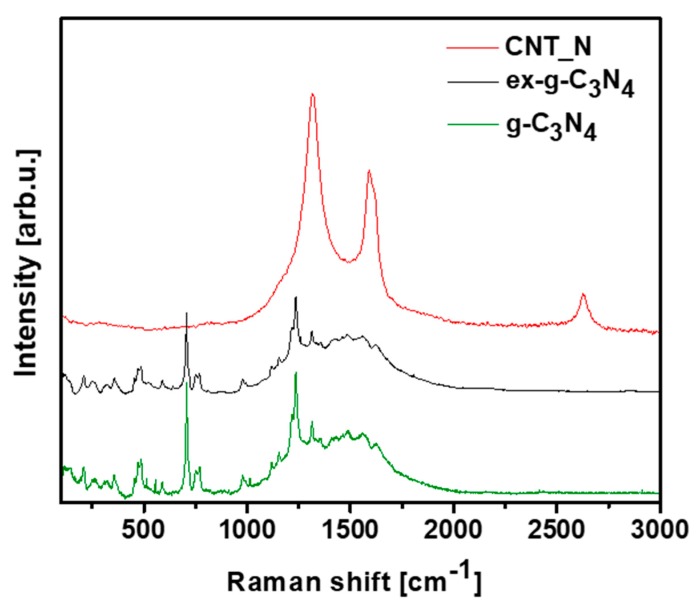
Raman spectra of g-C_3_N_4_, ex-g-C_3_N_4_ and CNT-N.

Raman spectroscopy was introduced to provide information about the chemical structures of g-C_3_N_4_, ex-g-C_3_N_4_ and CNT-N (Figure 11). Raman spectra of g-C_3_N_4_ and ex-g-C_3_N_4_ contain several bands observed in the range of 700–1630 cm^−1^ which are attributed to graphitic carbon nitride [40,41,42]. Additionally, the vibrations at 753, 977, 1120, 1156, 1236 and 1314 cm^−1^ are assigned to the stretching vibration of aromatic C-N heterocycle characteristic to melem. [43,44] The peaks at 700–1000 cm^−1^ are related to the different types of ring breathing modes of s-triazine. The Raman response of the tubular sample exhibits D (1318 cm^−1^), G (1580 cm^−1^) and 2D (2700 cm^−1^) bands what confirm the formation of graphitic carbon structure in the sample [45,46].

### 3.3. Mechanism of the CNT-N Formation

**Figure 12:**
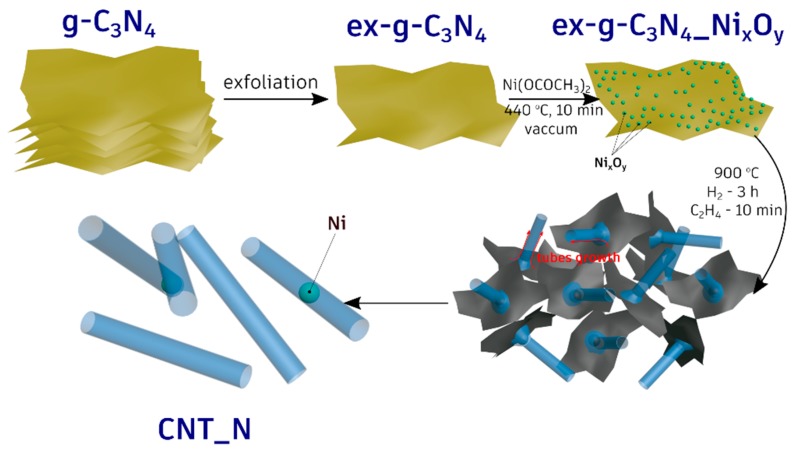
Schematic diagram of N-doped carbon nanotubes synthesis.

All the performed experiments clearly indicate that the metallic nickel serves as an active catalyst for the formation of the nanotubes. To verify this, the additional experiments in which g-C_3_N_4_-Ni_x_O_y_ were treated with hydrogen or ethylene at 900 °C were performed (see Figure 4 and Figure 5). The tubular forms did not evolve when g-C_3_N_4_-Ni_x_O_y_ was treated only with H_2_. Moreover, treating of g-C_3_N_4_-Ni_x_O_y_ only with ethylene (no hydrogen step) at 900 °C results in the formation of cocktail of carbon onion-like structures or short irregular tube-like structures. Therefore, the key step was the reduction of nickel oxides to metallic nickel in the hydrogen/ethylene flow. This was also confirmed by XRD studies (see Figure 4). In the next step, carbon and nitrogen from g-C_3_N_4_ diffused into nickel particles and when the oversaturation was reached these elements started to grow in the form of tubes. The elemental and the structural properties of the tubes were confirmed in EDX analysis and the Raman response of the sample. The whole process of the tubes formation is presented schematically in Figure 12.

## 4. Conclusions

In summary, we present a simple and effective method for the synthesis of N-doped carbon nanotubes with a diameter in the range of 200–300 nm. CNT-N were produced in the reaction of chemical vapor deposition in the presence of ethylene flow. Here, exfoliated graphitic carbon nitride functionalized with nickel oxides has been used as a starting material in CNT-N formation. This material plays a double role: (1) nickel was a catalyst in nanotube growth and (2) graphitic carbon nitride served as support for nickel nanoparticles and as carbon/nitrogen source. The results also prove that ethylene plays a crucial role in the formation of tubes. On one side, it carboreduced the nickel oxide (II) to metallic nickel which was an active phase in the growth of the nanotubes, and, on the other hand, it was a source of carbon in the final sample. The obtained N-doped nanotubes may show promising performance in a variety of applications such as catalysis, lithium-ion batteries, supercapacitors and so on.
